# P02.17. Building a database of validated pediatric outcomes: an investigation of compliance with established reporting standards

**DOI:** 10.1186/1472-6882-12-S1-P73

**Published:** 2012-06-12

**Authors:** D Adams, Y Liu, S Surette, L Hartling, S Vohra

**Affiliations:** 1 University of Alberta, Edmonton, Canada; 2 ARCHE, Dept of Pediatrics, University of Alberta, Edmonton, Canada

## Purpose

Many pediatric trials are published annually, but criticism exists regarding outcome measures used and their reporting. Reporting standards set out by the Consolidated Standards of Reporting Trials (CONSORT) group require accurately defined outcomes, and apply equally to studies of conventional or complementary and alternative medicine (CAM). The objective of this study was to conduct a systematic review to identify gaps in outcome reporting in pediatric randomized controlled trials (RCTs).

## Methods

Ten journals with the highest impact factors were searched for pediatric RCTs published between 2000-2010. Two independent reviewers conducted screening and data extraction on 20% of randomly selected included studies. Variables extracted included: journal, sample size, participant age, condition under study, intervention, control, and details of primary outcome and outcome measurement tools.

## Results

Searches identified 2229 unique references. Screening of a random sample of 2.5% determined that most (97%) were RCTs, thus full text for all references were obtained. Inclusion screening was carried out simultaneously with data extraction. Of the 446 articles screened to date, 66% were included. Participant age ranged from 20 weeks gestation to 20 years. Most (65%) were of treatment rather than prevention. Commonly used controls included placebo (35%) and another intervention (33%). With respect to primary outcome reporting, 34% of trials did not identify a primary outcome. Half (53%) reported at least one primary outcome; of these, 55% described one outcome as primary and 38% identified more than one outcome as primary. One quarter of the trials that included only one primary outcome used a questionnaire or scale-based tool and of these, only 26% presented information on tool clinometrics.

## Conclusion

This project will help identify gaps in the quality of outcome reporting in pediatric trials published in top journals over the past 10 years, leading to recommendations for improvements in reporting standards.

